# 

**Published:** 1911-03-18

**Authors:** 


### CONTENTS.

| | PAGE |
|---|---|
| **Mr. Shaw Again** | 699 |
| **Medical Defence** | 700 |
| **Annotations**— The Order of St. John—The Fees of Medical Witnesses—The Treatment of Medical Witnesses—The Medical Navy League | 701 |
| **Medical Opinion and Movement** | 702 |
| **Hospital Clinics**— Infective Arthritis.—I. By W. D'Oyly Grange, M.D. Edin. | 705 |
| **Special Articles**— I.—The Practice Market | 707 |
| Ionisation | 708 |
| **News and Events of the Week** | 709 |
| **Medical Appointments** | 710 |
| **Obituary** | 710 |
| **Parliament and Publications** | 710 |
| **"The Hospital" Medical Book Supplement.—No. XL.**— | 71 |
| **The Week's Work**— Great Ormond Street Hospital—Mrs. Bateman's Will—The Week's Drug Market... | 715 |
| **Metropolitan Hospital Sunday Fund** | 717 |
| **Hospital World** | 720 |
| **Gifts and Bequests** | 720 |
| **Jottings** | 720 |
| **The Hospital for Sick Children, Great Ormond St.** | 721 |
| **News and Events of the Week** | 724 |
| **Buildings Contemplated and in Progress** | 724 |
| **Appointments Vacant** | 724 |

